# Recording mobile DNA in the gut microbiota using an *Escherichia coli* CRISPR-Cas spacer acquisition platform

**DOI:** 10.1038/s41467-019-14012-5

**Published:** 2020-01-07

**Authors:** Christian Munck, Ravi U. Sheth, Daniel E. Freedberg, Harris H. Wang

**Affiliations:** 1 Department of Systems Biology, Columbia University, New York, NY USA; 2 Integrated Program in Cellular, Molecular, and Biomedical Studies, Columbia University, New York, NY USA; 3 Department of Medicine, Columbia University, New York, NY USA; 4 Department of Pathology and Cell Biology, Columbia University, New York, NY USA

**Keywords:** Microbial communities, Bacterial genes

## Abstract

The flow of genetic material between bacteria is central to the adaptation and evolution of bacterial genomes. However, our knowledge about DNA transfer within complex microbiomes is lacking, with most studies of horizontal gene transfer (HGT) relying on bioinformatic analyses of genetic elements maintained on evolutionary timescales or experimental measurements of phenotypically trackable markers. Here, we utilize the CRISPR-Cas spacer acquisition process to detect DNA acquisition events from complex microbiota in real-time and at nucleotide resolution. In this system, an *E. coli* recording strain is exposed to a microbial sample and spacers are acquired from transferred plasmids and permanently stored in genomic CRISPR arrays. Sequencing and analysis of acquired spacers enables identification of the transferred plasmids. This approach allowed us to identify individual mobile elements without relying on phenotypic markers or post-transfer replication. We found that HGT into the recording strain in human clinical fecal samples can be extensive and is driven by different plasmid types, with the IncX type being the most actively transferred.

## Abstract (web summary)

Horizontal gene transfer (HGT) in complex bacterial communities has been mostly studied using metagenomic analyses. Here, the authors develop an *E. coli* CRISPR-Cas spacer acquisition platform that allows real-time recording of HGT events at nucleotide-resolution, identifying diverse DNA transfer events in human clinical fecal samples.

## Introduction

Densely populated polymicrobial communities exist ubiquitously in natural environments such as soil and the mammalian gastrointestinal tract. Bacteria in these microbiomes are thought to engage in extensive horizontal gene transfer (HGT) based on metagenomic sequencing studies and comparative genomics analyses^1–4^. HGT is a natural phenomenon where DNA is exchanged between organisms through distinct mechanisms including cell-to-cell conjugation of mobile plasmids or genetic elements, transduction by phages and viruses, or transformation by uptake of extracellular nucleic acids^5^. Upon horizontal transfer, the foreign DNA can be either retained in the recipient or lost over time. HGT processes play a driving role in the evolution of bacterial genomes, leading to the dissemination of important functions such as complex carbohydrate metabolism^6^, pathogenicity^7^, and resistance to antibiotics^8^ or toxic compounds^9^.

Despite the prevalence of HGT, the evolutionary selection that drives fixation of foreign DNA is generally not well understood; for example, roughly 30% of genes predicted to be acquired by HGT have no known function^3^, and pan-genome analysis of sequenced genomes predict that many species have open-ended pan-genomes with enormous potential for gene turnover^10–12^. For fixation of transferred DNA to occur in recipient cells many barriers must be overcome, such as specific selection pressures, fitness burden of the acquired element, genetic compatibility with host machinery (e.g., replication, transcription, translation) and presence of anti-HGT systems such as restriction modification systems or CRISPR-Cas systems^5,13,14^. In addition, the presence of addiction elements on the transferred DNA (e.g., toxin–antitoxin and partitioning systems) also influence the fate of the transferred element. Even when the transferred genetic element provides a fitness benefit they may require many generations to be fixed in a population^15^. The architecture and dynamics of these gene-flow networks are often not known, especially since most HGT genes are identified from endpoint analyses.

Contemporary computational methods for inferring HGT events rely on different approaches including identification of shared mobile elements such as plasmids or phages, analysis of genomic abnormalities (e.g., shifts in GC% or codon usage) or phylogenetic comparisons between a candidate gene and a conserved gene (e.g., 16 S rRNA)^16^. On the other hand, experimental approaches to study HGT require the transferred DNA to confer a detectable phenotype that can be enriched in the population. However, not all mobile elements confer a readily selectable phenotype. New selection-independent methods that can capture real-time transfer dynamics across a population will provide a deeper and richer understanding of the overall HGT process.

As a consequence of the pervasive gene flow in microbial genomes, bacteria have evolved various defense systems to manage horizontally acquired genetic material^5,17^. CRISPR-Cas systems can provide specific and adaptive immunity to invading DNA^13,14^. During the conserved CRISPR adaptation process, Cas1 and Cas2 proteins capture short fragments of invading DNA and integrate them as spacers into CRISPR arrays^13,18^, a process that requires active cell division^19^. In *E. coli*, immunity is conferred by transcribed spacers guiding the CRISPR-associated complex for antiviral defense (Cascade) to the invading DNA^20^. Importantly, the CRISPR arrays provide a useful long-term record of horizontally invading DNA.

Different CRISPR-Cas types have been identified across bacterial and archaeal phyla and have been engineered to study spacer adaptation^21^. Adaptation of new spacers into CRISPR arrays is a rare event under simulated natural conditions^13,22^, and in contrast to acquired immunity in *Streptococcus thermophilus*^13^, most natural *E. coli* strains do not actively acquire new spacers and their arrays therefore reflect ancient HGT events^23^. However, spacer acquisition can be stimulated if the CRISPR array is ‘primed’ with a spacer matching the foreign DNA^24^. Furthermore, heterologous expression of *cas1* and *cas2* can lead to high levels of spacer adaptation^24,25^, a process that can be leveraged for engineered signal recording and storage applications^26–28^.

Here, we leverage the CRISPR spacer acquisition process as a mechanism for real-time recording of HGT events at nucleotide-resolution. Using an optimized acquisition system, we can capture transient HGT events and identify DNA transfers that cannot be easily detected with traditional methods. The performance and technical accuracy of this system was rigorously characterized using defined donor strains and communities. Application of the system to clinical human fecal samples revealed prevalent and diverse DNA transfer events, shedding light on the dynamics of HGT in the mammalian gut microbiome into an *E. coli* recipient.

## Results

### Identifying exogenous HGT using CRISPR spacer acquisition

**Fig. 1:**
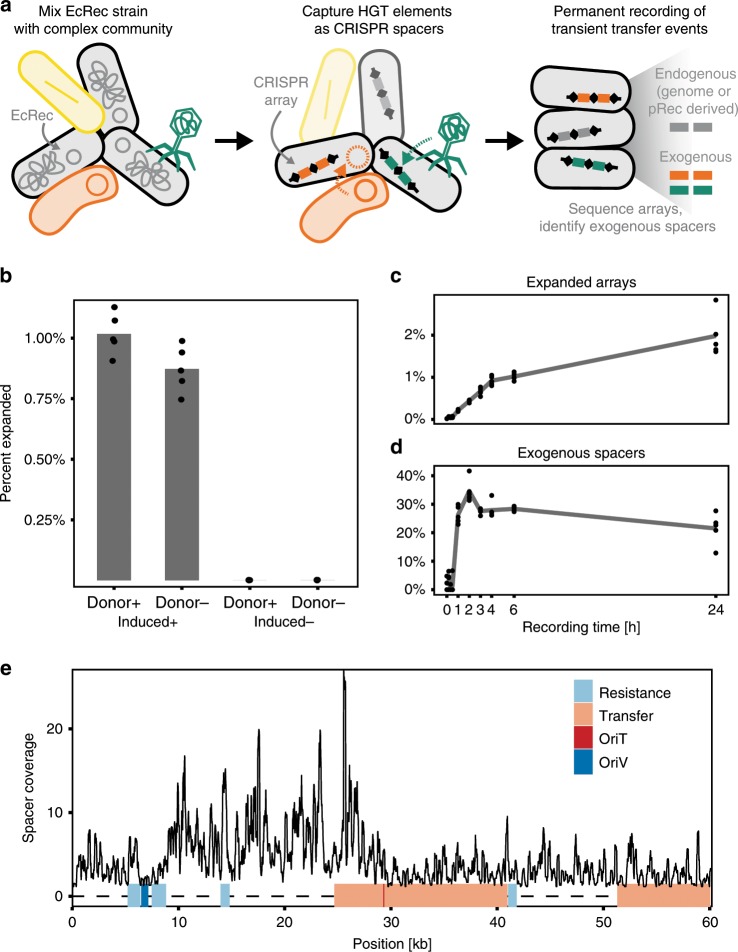
Recording HGT with engineered CRISPR acquisition. **a** Schematic of HGT recording where the EcRec strain is mixed with donor cells and spacers are acquired from both endogenous and exogenous DNA sources. Resulting CRISPR arrays are sequenced to determine the identity and origin of spacers. **b** Results from recording for 6 h with or without induction and with or without FS1290/RP4 as donor strain (*n* = 5 *biological replicates* with mean bar, no gel-extraction). **c** Array expansion is detected within 1 h after induction and increases rapidly for the first 4–6 h (*n* = 5 *biological replicates* with mean line, no gel-extraction). **d** Unique exogenous spacers are detected 1 h after induction constituting ~30% of all spacers (*n* = 5 *biological replicates* with mean line, no gel-extraction). **e** Mapping of recorded unique spacers to the RP4 plasmid. Spacer coverage is average coverage per bp. in 200 bp. windows and based on 5 *biological replicates*.

We previously engineered a CRISPR-based temporal recording system that acquired new spacers from either endogenous genomic DNA or a copy-number inducible plasmid^28^. In this system, we utilized a recording strain (hereafter referred to as EcRec) consisting of *E. coli* BL21 with the pRec-*ΔlacI* plasmid containing an anhydrotetracycline (ATc) inducible operon of the *E. coli* Type I-E *cas1* and *cas2* genes. Upon induction of the recording strain, over-expressed Cas1 and Cas2 proteins incorporate DNA protospacer sequences into CRISPR array I on the genome at high frequencies^28^. Since *E. coli* BL21 lacks the Cascade interference machinery, acquired spacers do not lead to CRISPR-mediated adaptive immunity^20^. The system can thus serve as a recorder of intracellular DNA. CRISPR expansions can be easily analyzed by PCR amplification of the CRISPR array from a population of recording cells, and, if needed, enrichment for arrays with new spacers can be achieved by a simple gel extraction of expanded array products. Subsequent deep amplicon sequencing can be used to assess the spacer repertoire^28^. While spacers can be acquired from both endogenous and exogenous DNA sources, including the genome, there is a strong preference to acquire spacers from high copy replicative plasmids^19,22^. Given the capacity of the *cas1/cas2* overexpression system to record intracellular DNA at much higher efficiency than the wild-type system, we hypothesized that the system could be used as a sensitive method to reveal HGT events (Fig. 1a) that may only occur transiently or at a low-frequency across a cell population.

To explore whether CRISPR recording can allow direct measurement of HGT events, we exposed the recording strain (EcRec) to the *E. coli* strain FS1290 that harbors the well-characterized broad host range conjugative plasmid RP4 ref. ^29^. Before mixing the two strains, expression of *cas1* and *cas2* was induced to ensure maximum acquisition capacity (see Methods). In addition, non-induced EcRec served as a control. Conjugation was carried out by mixing the strains in a 1:1 ratio and spotting them on agar plates with and without ATc. Reactions without the donor *E. coli* FS1290 strain served as an additional control. After 6 h, the cells were collected and CRISPR arrays were amplified and sequenced (without gel extraction) to evaluate the spacer repertoire, yielding 10^4^–10^5^ sequenced arrays per biological replicate (Supplementary Data 1). In the *cas1/cas2* induced cells with donor, 1.0% (sd = 0.1%, *n* = 5 recordings) of the arrays were expanded in contrast to only 0.0010% (sd = 0.0006%, *n* = 5 recordings) in the non-induced cells (Fig. 1b). Further probing of the dynamics of the HGT recording process showed that overall spacer expansion could be identified as early as 1 h after mixing the donor and recording cells, with the rate of array expansion leveling off after 4 to 6 h of conjugation (Fig. 1c). By 24 h, 1.9% of all arrays (sd = 0.5%, *n* = 5 recordings) were expanded (Supplementary Data 1).

As expected, most spacers were derived from the EcRec genome and pRec plasmid. We therefore applied a stringent two-step filter against a de novo sequenced EcRec/pRec reference to isolate putative exogenous spacer sequences. First, only spacers flanked by the canonical direct repeat sequences were kept. Second, spacers with even moderate sequence homology (≥80% identity and coverage) to the EcRec genome or the pRec plasmid were removed (and Methods and Supplementary Methods). Using these filtering criteria, we found that among the expanded arrays, exogenous spacers constituted up to 30–40% of all new spacers and could be detected within 1 h of conjugation (Fig. 1d). After 24 h, 21% (sd = 5%, *n* = 5 recordings) of the sequenced spacers were identified as exogenous. The number of exogenous spacers was influenced by the ratio of donor to recording cells, and we could detect new exogenous spacers in as few as 1 donor per 10^6^ recording cells (Supplementary Fig. 1). In comparison, only 0.5% (sd = 0.2%, *n* = 5 recordings) of the spacers in the induced no-donor experiment were identified as exogenous, likely representing spacer sequences containing technical sequencing errors (Supplementary Data 1).

In complex microbiomes, the identity of potential transferred elements is unknown. However, acquired exogenous spacers can be matched against large sequence databases (e.g., GenBank refseq) to identify specific mobile elements. To define the criteria for a match between a spacer and a reference database, we first gel extracted and sequenced spacers from the 24-h *E. coli* FS1290 recording samples (Supplementary Data 2). Then, a set of scrambled spacers was generated by randomly reordering the sequence of the exogenous spacers. Using BLAST, both original and scrambled spacers were searched against the Genbank RefSeq bacterial genomes database. We identified a conservative hit-threshold of ≥95% identity and coverage that prevented spurious matches of scrambled spacers to the database (Supplementary Fig. 2). Using this threshold, we found that 98.6% (sd = 0.2%, *n* = 5) of the unique exogenous spacers could be mapped back to the RP4 plasmid sequence (Fig. 1e) and that spacers were acquired across the plasmid, preferably from sites corresponding to the known PAM recognition sequence of *E. coli* Cas1/Cas2 (AAG, 50% of all spacers, Supplementary Fig. 3). In addition, we observed increased spacer density between the origin of replication (oriV) and the origin of transfer (oriT) (Fig. 1e). Early work on spacer adaptation has shown that acquisition hotspots exist^30^ and it has been shown that spacer adaptation is a replication dependent process with increased spacer acquisition at stalling replication forks^19^. We speculate that single stranded nicks at the oriT site stall the unidirectional replication fork of RP4 ref. ^31^ causing increased spacer adaption. Together, these results show that the EcRec is capable of recording DNA transfer events robustly with high sensitivity and that exogenous spacers can be confidently mapped to the mobile DNA of origin.

### Detection of non-replicative and complex HGT events

**Fig. 2:**
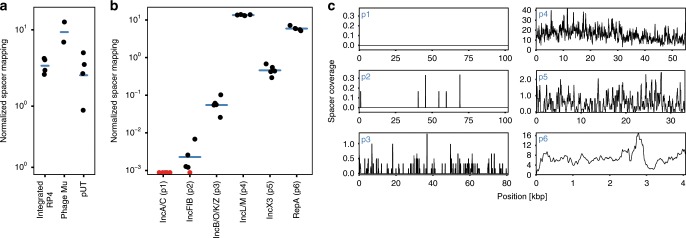
Detecting non-replicative and complex HGT events. **a** EcRec acquires spacers from transferrable but non-replicating DNA elements in E. coli S17, the integrated RP4, phage Mu and the non-replicating plasmids pUT. In total 77,825 spacers were obtained. The normalized spacer mapping is spacers per kb per 1000 exogenous spacers. (*n* = 4 biological replicates with mean bar, recorded for 6 h). **b** Recorded spacers from Ec70 carrying six plasmids (p1–p6). For each plasmid the number of matching spacers is normalized as spacers per kb per 1000 exogenous spacers. Red data points denote zero recorded spacers. No spacers are recorded from plasmid p1 (*n* = 5 biological replicates with mean bar, recorded for 6 h). **c** Mapping of recorded spacers to the plasmid sequences, substantial coverage is seen for all plasmids except the large plasmids p1 and p2 (spacer coverage is average coverage per bp. in 200 bp. windows, based on 5 biological replicates).

Many HGT events may be transient or may occur at low frequencies. We hypothesized that our recording system could capture spacers from HGT events in which the transferred element is not stably maintained in a recipient. To investigate transfer of both genomic DNA and a non-replicative plasmid we used an *E. coli* S17 strain carrying the R6K-derived plasmid pUT, as the donor^32^. *E. coli* S17 contains a genomically integrated copy of the RP4-Tet::Mu conjugation system and also expresses the R6K replication initiation protein Pir. The integrated RP4 can mobilize the S17 genome and the pUT plasmid into recipient cells^33^. However, pUT requires the Pir protein *in trans* in order to replicate and therefore cannot be maintained in the EcRec recipient, which lacks the *pir* gene^32,34^. In addition, phage Mu, which is also present in S17, can be acquired by recipients either via conjugation of the S17 genome or via phage particles^34^. We mixed EcRec with the *E. coli* S17/pUT donor strain and recorded spacers for 6 h. Analysis of new exogenous spacers from the S17/pUT donor showed acquisition from both the integrated RP4-Tet::Mu and the pUT plasmid, highlighting that active replication of the transferred element is not required for spacer acquisition (Fig. 2a). We further investigated whether EcRec could record DNA from infecting M13 phage. As M13 infects cells via the F-pili we first generated a phage susceptible EcRec by conjugating F′ from *E. coli* K603 into EcRec. Induced EcRec/F′ was exposed to M13 and after 24 h incubation with ATc, and the arrays were amplified and sequenced. We found six spacers acquired from M13 (Supplementary Fig. 4), showing that recording of this phage was possible although with a low efficiency. Previous work studying spacer acquisition from infecting M13 in Cascade proficient *E. coli* found a 3% acquisition rate amongst isolates selected for their resistance to phages using a multiplicity of infection (MOI) of 10 ref. 24. The low adaptation rate in our study, even in the background of *cas* over expression, may be caused by the reduced growth rate the infected cells^35^. We also attempted to detect transfer of the Gram-positive mobile plasmids pGO400 and pSL20 from *S. aureus* and *B. subtilis*, respectively, but we could not detect any spacers. We speculated that this might be due to the plasmids not entering the recording cells. To test this, we electroporated the plasmid DNA into the recording cells, in these cases we were able to detect spacers at low frequencies from electroporated plasmid as well as *S. aureus* and *B. subtilis* chromosome (Supplementary Fig. 5).

Since natural bacterial isolates often carry multiple plasmids capable of transfer, we tested if our recording system could resolve transfer of different mobile elements from the same donor. A clinical *E. coli* isolate (Ec70) that carried 6 different plasmids (p1–p6), as resolved by hybrid assembly (Oxford Nanopore and Illumina sequencing, Methods), was used as the donor strain (Supplementary Data 3). Sequencing and analysis of new spacers from a recording experiment with Ec70 revealed that 97% of exogenous spacers were acquired from only two plasmids, the 55 kb plasmid p4 and the 4 kb plasmid p6. We quantified the spacer mapping to the reference sequence as the average number of spacers per kb per 1000 exogenous spacers, hereafter referred to as normalized spacer mapping (Fig. 2b). The normalized spacer mapping of each plasmid provides a semi-quantitative estimate of its relative transfer frequency, however, variations in PAM-site frequency and plasmid copy number will affect the estimates.

For p4 and p6 the normalized spacer mapping was 13.5 (sd = 0.4), and 6.0 (sd = 0.8), respectively (*n* = 5). While plasmid p4 is self-transmissible, the much smaller plasmid p6 only carries the mobilization protein MobA, hence requiring the conjugation apparatus *in trans*. Neither of the two plasmids carry any antibiotic resistance genes highlighting that our recording system can readily detect elements that would not be easily detectable by standard selection-based methodologies. Plasmids p3 and p5 (80 kb and 33 kb, respectively) appeared to transfer, although at very low frequencies with a normalized spacer mapping of 0.060 and 0.48, respectively (sd = 0.03 and 0.15, *n* = 5 recordings). No spacers were observed from the 106 kb large plasmid p1 and only eight spacers were observed from the 102 kb plasmid p2 (Fig. 2b) from a total of ~1 million expanded spacers. As expected, spacers were acquired from across the plasmid backbones (Fig. 2c). These results demonstrate that CRISPR-based recording of HGT can reveal and resolve the transfer dynamics of different mobile elements from a donor carrying 6 plasmids.

### Capturing HGT events from a defined microbial community

**Fig. 3:**
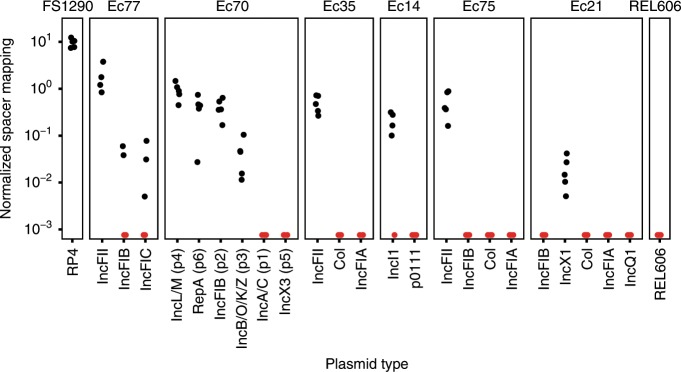
Recording of HGT events in a defined multispecies community. Spacer recording in a defined community of 8 *E. coli* strains. Exogenous spacers (*n* = 14,463 pooled over 5 biological replicates) were mapped to contigs identified as plasmids^36^ in the 8 genomes allowing only unique hits. Hits were observed for all donors except the negative control REL606, which carries transferrable genetic elements. The normalized spacer mapping is spacers per kb per 1000 exogenous spacers. Red data points denote zero recorded spacers.

Having characterized the recording system using a single donor, we explored whether HGT events could be recorded in a complex, multi-donor community. A defined bacterial community comprised of 6 clinical *E. coli* isolates (Ec77, Ec70, Ec35, Ec14, Ec75, Ec21) as well as a positive control strain (FS1290) that carries the RP4 plasmid, and a negative control strain (REL606) that contains no plasmids was assembled. We generated draft genome assemblies and predicted that the clinical *E. coli* strains carried at least two plasmids each, including Ec70 already established to carry six plasmids^36^ (Fig. 3, Supplementary Data 3).

Donor strains were pooled in equal ratios and then mixed with EcRec. The recording was carried out for 6 h on LB agar + ATc and new exogenous spacers were identified and mapped back to the contigs from the draft genome assemblies for each of the 7 donor strains while the hybrid assembly was used for Ec70 strain. Spacers mapping to more than one contig were filtered out to ensure an unambiguous interpretation of HGT events (26.0%, *n* = 3205). We detected new spacers from all donor strains except from the negative control REL606 (Fig. 3). However, spacers were not acquired equally from the donors, with 72% (sd = 9%, *n* = 5 recordings) of all spacers deriving from the FS1290 positive control strain, confirming that RP4 transfers at high frequency^37^. Clinical strains Ec77 and Ec70 were particularly efficient donors, representing 19% and 7.3% of total spacers, respectively (sd = 9% and 2.4%, *n* = 5 recordings). Based on this mapping, we could also identify which predicted plasmids were being transferred. For instance, IncFII-type plasmids in Ec35, Ec75, Ec77 appear to transfer readily to EcRec, while col-type plasmids in Ec21, Ec75, and Ec77 do not appear to mobilize. Importantly, we qualitatively detect the same transfer profile for Ec70 in this community recording as in the single donor recording. However, all spacers mapping to the IncX3 plasmid in Ec70 were removed due to redundant mapping to other plasmids in the community.

### Capturing HGT events from natural microbial communities

**Fig. 4:**
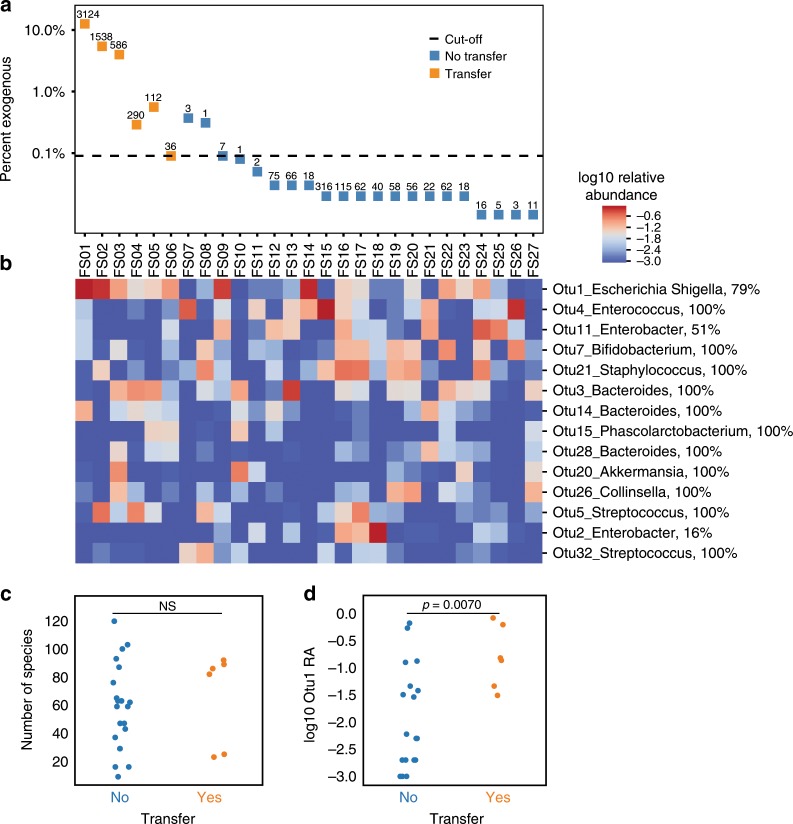
Measurement of HGT events in 27 clinical fecal samples. **a** Identifying fecal samples with robust exogenous spacer acquisition. Percent of spacers classified as exogenous for each sample. Samples with at least 0.09% exogenous spacers (dashed threshold line) and a minimum of 10 unique exogenous spacers (denoted above each data point) were classified as samples with HGT events (orange data points). **b** Cluster map of 16 S operational taxonomic units (OTUs) abundance for the 27 fecal samples. Samples with observable transfer (FS01-FS06) or no observable transfer (FS07-FS27) are shown. OTUs observed at >0.05 relative abundance in at least 2 samples are shown; log10 relative abundance is displayed. For each OUT the predicted species is noted with the confidence of the prediction (%). **c** Number of unique OTUs per samples stratified by transfer status. **d** Relative abundance of Otu1 (*Escherichia/Shigella*) stratified by transfer status. Samples with transfer have a significant higher abundance of *Escherichia/Shigella* (*p* = 0.0070, Mann–Whitney U test).

Extensive HGT has been reported in the human microbiome and has been shown to facilitate the spread of clinically important genes such as antibiotic resistance genes^3,4,38–40^. Therefore, we sought to identify mobile DNA accessible to *E. coli* in clinically relevant human fecal microbiomes. Fecal samples were from hospitalized adults with diarrhea whose stools were tested for *Clostridium difficile* infection (CDI). Of 27 patient-samples, 24 had received broad-spectrum antibiotic treatment in the month prior to sampling while the remaining (FS05, FS06, and FS07) did not receive antibiotics. For each sample, ~0.5 g of fecal matter was washed in PBS three times to remove potential inhibiting compounds, such as antibiotics. The washed samples were each mixed with pre-induced EcRec, spotted on LB agar + ATc, and incubated for 24 h. In order to confidently identify samples with HGT events we established strict criteria requiring that at least 10 exogenous spacers were identified and that the percentage of exogenous spacers was at least 3 times higher than in the no-donor control samples (0.03%). From the 27 recordings, we sequenced >10 million CRISPR arrays (Fig. 4a). Six recordings passed our criterion representing a total of 20,991 exogenous spacers yielding 5686 unique spacers (Supplementary Data 2). To investigate the reproducibility of the fecal recordings, we repeated the recording for three samples that had a high number of exogenous spacers (FS01-03) and three samples with a low number of exogenous spacers (FS10-12). We found that the overall within-sample reproducibility was high and that the samples were clearly delineated into the initial groups of high and low numbers of exogenous spacers (Supplementary Fig. 6). Furthermore, we investigated how sequencing depth affected the number of exogenous spacers and we saw no correlation between sequencing depth and the number of exogenous spacers within the six samples with HGT events (Supplementary Fig. 7).

We hypothesized that the presence of closely related donor species would be important to observing HGT. We thus profiled the composition of all 27 fecal samples using 16 S rRNA amplicon sequencing (Fig. 4b). Overall, the α-diversity (number of species) was similar regardless of whether or not high numbers of exogenous spacers were observed (Fig. 4b, c). However, as predicted, we found that the relative abundance of the *Escherichia/Shigella* taxa (Otu1) was significantly elevated in the 6 samples passing the recording criteria (Fig. 4d, *p* = 0.0070, two sided Mann–Whitney U test). Still, some samples with high abundance of *Escherichia/Shigella* had few exogenous spacers (e.g., FS09 and FS14), suggesting that presence of *Escherichia/Shigella* at high abundance is correlated with but not sufficient for detectable transfer (e.g., presence and mobilization of plasmids may be variable or bacteriocins production might inhibit EcRec).

**Fig. 5:**
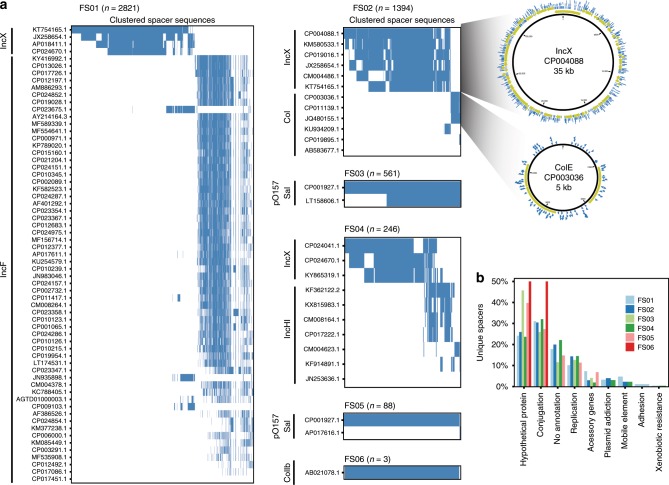
Analysis of human-associated mobilome from HGT recordings. **a** Exogenous spacers mapped to the custom plasmid database, each row represents a plasmid (denoted by accession number). The mappings are filtered to include the fewest number of plasmids covering all spacers. Rows are sorted in order of the number of spacers that map to the plasmid. The sorting enables easy identification of discrete transferred elements. Each spacer cluster is annotated with the predicted plasmid group based on Plasmid Finder^36^. Spacer mapping is illustrated for FS02 showing the plasmid backbone with predicted open reading frames (ORFs) (yellow) and mapping unique spacers (blue). **b** Annotation categories overlapping with the spacers from all six clinical recordings. Genes predicted to be involved in conjugative transfer dominate, followed by unannotated genes and genes involved in plasmid replication. Notably, very few spacers overlap with genes involved in drug resistance.

To identify the source of exogenous spacers, we used BLAST to search the NCBI RefSeq bacterial genome database, NCBI RefSeq viral genome database and a custom plasmid database applying the previously established thresholds (Methods). Overall, the majority of the 5686 unique exogenous spacers could be matched to at least one of the databases (Supplementary Data 2). All spacers with hits to the viral database also matched to the genome database. Furthermore, > 95% of spacers with hits to the genome database also matched to the plasmid database, highlighting that the identifiable spacers were most likely of plasmid origin. For each sample, we identified the minimal set of reference plasmids that encompass all spacers. Clustered heatmaps from these plasmid hits were used to identify the likely source plasmid of the exogenous spacers and predict the number of discrete mobile genetic elements recorded from the sample. For each sample, we infer that 1–2 different plasmids were transferred (Fig. 5a).

For instance, BLAST hits of spacers to the plasmid database in sample FS02 indicate that two plasmids were transferred, a large IncX-type plasmid and a small colE-type plasmid (Fig. 5a). The putative IncX hits match to a 35 kb plasmid (Genbank accession CP004088) carrying no resistance markers. The acquired spacers map across the entire plasmid back-bone, suggesting that the reference is a good representation of the transferred plasmid. The small colE-like plasmid (Genbank accession CP003036) has three predicted open reading frames (ORFs): a replication protein, a mobilization protein and an unknown ORF. While spacer coverage of the colE plasmid is sparser than the IncX plasmid, spacers matched across the back-bone suggesting that all regions of the pCE10B plasmid were present in the mobilized plasmid captured from FS02. Interestingly, the smaller plasmid does not encode a conjugation apparatus and therefore requires conjugation genes *in trans* for mobilization. Mapping all acquired spacers to the Plasmid Finder database^36^ revealed matches to IncX, IncI, IncF, IncH, pO157_Sal, and colE plasmid types, which are all common replicons in *Enterobacteriaceae* (Fig. 5a).

To better delineate the functions of the ORFs that yielded spacers, we used the RefSeq database to extract the functional annotations of genes with spacer hits (Fig. 5b and Supplementary Data 4). For each sample, 80–85% of spacers had functional annotations. The most common gene annotations were associated with canonical plasmid functions including conjugation, replication and plasmid addiction genes. As expected, a large portion of the ORFs had no known function (Fig. 5b). Given that the majority of patients received antibiotics recently (4/6 with detectable transfer), one might expect that the transferrable plasmids would harbor antibiotic resistance genes. However, mapping of spacers to the ResFinder database^41^ yielded only two spacer hits to antibiotic resistance genes, a bla_TEM_ beta-lactamase and a chloramphenicol acetyltransferase gene (both from FS04), suggesting that, although present, resistance genes are not particularly abundant in the pool of mobile plasmid that can move into EcRec, even after extensive antibiotic treatment.

### Identification of transferred plasmids from metagenomes

**Fig. 6:**
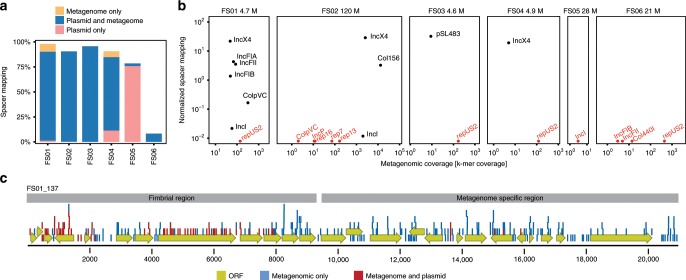
Metagenomic verification of predicted transfer events. **a** Percentage of spacers that could be mapped to the metagenomic contigs only (yellow), plasmid database and metagenomic contigs (blue), or plasmid database only (pink). **b** Mapping of spacers to predicted metagenomic plasmid contigs as a function of contig coverage in the assembly. The normalized spacer mapping is spacers per kb per 1000 exogenous spacers. Red data points denote zero recorded spacers. Number above each plot denotes the number of reads in the metagenome (millions). **c** Contig from FS01 where the majority of spacers were specific to the metagenome (blue). The contig consists of a region encoding a P-type fimbria and a region containing most hypothetical proteins specific to the metagenome.

We further performed shotgun metagenomic sequencing on the original fecal samples to assess the recovery of spacers against assembled contigs and to confirm the presence of putative plasmids in the samples. Metagenomic reads were assembled yielding ~371 Mbps of contigs across the six samples with observed plasmid transfer events (FS01-FS06) (Supplementary Data 5). Most acquired spacers could be matched to metagenomic contigs by BLAST (Fig. 6a). However, in two samples, FS05 and FS06, the metagenomic recovery rate was very low (3% and 8%, respectively). Correspondingly, these samples also had few acquired unique exogenous spacers (112 and 36, respectively), suggesting low frequency of HGT. Mapping of all exogenous spacers to the plasmid database revealed that the majority of spacers matched to both metagenomic contigs and published plasmids, confirming that most transfer was via plasmids (Fig. 6a).

Using the Plasmid Finder database^36^, we identified putative plasmid contigs across the metagenomes. We observed transfer of a variety of *Enterobacteriaceae* plasmids including IncF, IncX, IncI, and col types, corroborating the results generated with our plasmid database (Fig. 6b). In addition, we also detected a number of non-transferred plasmids (e.g., repUS2) from Gram-positive species including *S. aureus*. Interestingly, certain plasmid types appeared to transfer more readily than others based on comparing their spacer mapping density and metagenomic coverage. In particular, IncX-type plasmids transferred efficiently since we observed similar spacer mapping densities across three orders of magnitude in metagenomic coverage (Fig. 6b, FS01, FS02, and FS04). In contrast, IncI-type plasmids transferred at very low levels despite the metagenomic coverage varying two orders of magnitude between FS01 and FS02 (Fig. 6b).

In some cases, spacers mapped only to the metagenomic contigs (and not to the plasmid database; Fig. 6a and Supplementary Fig. 8). Among those contigs, contig 137 (21 kbp) from FS01 had a majority of metagenome-only spacers (202/276) indicating that the contig was not normally found on plasmids (Fig. 6c). This contig consisted of a region encoding a P-type fimbria along with a transposase as well as a region containing hypothetical proteins. The former region has been found in other plasmids, as indicated by spacer mapping to the plasmid database, while the latter region appears to be specific to the FS01 sample (Fig. 6c). The contig is not classified as plasmid, however, it is likely an incomplete assembly of a larger plasmid. Online BLAST of the contig against the nt database confirmed that there were no hits with broad coverage, and the best hit was an unnamed *E. coli* plasmid with 27% coverage at 92% identity to contig 137. This highlights the utility of our approach to identify novel transferred elements that may not be predicted by traditional reference-based methodologies.

## Discussion

Comparative analyses of sequenced genomes have provided important insight into the HGT processes^3,4^ occurring in different complex environments^3,4^. Our CRISPR-based recording system adds more detailed insights into the dynamics of HGT in complex environments by detecting DNA transfer events as they occur, enabling detection of transient transfer events.

The recording system captures spacers from HGT events stably into genomic arrays that can be used to identify mobile elements beyond current methodologies. The ability to detect, in real-time, transient transfer events and those occurring at low frequencies enables an in-depth characterization of mobile DNA in complex microbiomes.

As DNA transfer is confined by recipient range we are only capable of recording DNA that can enter the *E. coli* BL21 recording strain. When using other *E. coli* strains as donor we showed that transfers can be resolved down to the individual plasmids from donors that can carry up to 6 putatively mobile plasmids. We find that the different plasmids varied in transfer efficiencies likely reflecting differences in intrinsic plasmid transfer efficiency, donor transfer efficiency, recipient receiving efficiency, or a combination of these factors. Even though the observed mobile elements were all classified as plasmids, we still expect that phages are an important contributor to HGT. However, as illustrated by the recording of phage M13, our system is not optimal for detecting invading phages as CRISPR spacer adaptation requires active growth of the recording cell^19^, which is often impaired upon phage infection. In addition, infection with phages can lead to cell lysis and subsequent loss of recording cells from the population. Furthermore, it is not clear what the expected concentration of *E. coli* targeting phages is in human fecal samples. Previous studies indicate that plaque forming units (pfu) from human feces on *E. coli* is in the range of 10^1^–10^7^ per gram, with a median concentration of ~10^3^ per gram^42,43^. Considering that the concentration of *E. coli* in human feces is in the range of 10^7^–10^9^ per gram^44^, interactions between phages and the recording strain might be substantially less common than interactions between commensal *E. coli* and the recording strain. Washing of the fecal sample (i.e., to remove antibiotics and other factors that can inhibit the recording strain) likely also contributed to the loss of phage particles. Lastly, as *E. coli* is not naturally competent, we are not able to detect ‘naked’ environmental DNA. Consequently, our recording strain is best suited for detecting plasmid transfers.

When our approach is applied to clinical fecal specimens, we were able to identify active DNA transfer in 22% of the samples (6 out of 27). It is difficult to evaluate whether the frequency of observed HGT in the fecal samples (22%) is limited by the methodology or whether it reflects the enterobacterial transfer capacity of these samples. As the fecal recordings were performed aerobically, our approach was only able to capture transfers from aerobically active donors, potentially limiting some transfers. Additionally, the potential presence of bacteriocin producing strains could limit the growth of EcRec and hence lead to fewer acquired spacers. Yet, across the six metagenomes, the number of different plasmid replicons present varied greatly (Fig. 6b), suggesting that some samples contained fewer plasmids. In many instances, we observed multiple discrete plasmids being transferred, most of which did not carry selectable markers such as antibiotic resistance genes indicating that a substantially larger pool of active and mobile plasmids exists in the gut microbiome beyond just the antibiotic resistance plasmids that are typically identified by phenotypic assays.

By analyzing the captured spacers, we also found that many horizontally acquired genes have no known function, in agreement with previous bioinformatic analyses^3^. Using metagenomic sequencing, we definitively matched acquired spacer sequences to assembled plasmid contigs and plasmid types involved in these HGT events. While many different plasmids were identified in the metagenome, only subsets were shown to mobilize into EcRec, with the IncX type transferring most efficiently.

In the current system, spacer acquisition is driven by overexpression of *cas1* and *cas2*, yet after 24 h induction only about 2% of arrays are expanded, limiting the recording capacity and sensitivity of the system. Increasing the array expansion rate would improve the spacer output relative to sequencing depth and help improve sensitivity. However, the vast majority of spacers are acquired from endogenous sources, and it would therefore be desirable to increase the ratio of exogenous spacers to total spacers. Including an active Cascade complex could help counter select endogenous spacers, although, as many arrays adapt multiple spacers, it could potentially affect recording sensitivity.

Endogenous or engineered Cas1/Cas2 recording systems could be implemented in the context of different hosts to understand the host specificity of transfer for diverse bacterial species. These various systems and hosts could be multiplexed for high-resolution recording of HGT in various environments, from the human gut to various environmental microbiota. This would enable real-time recording of previously difficult-to-record transient HGT events, and offers a powerful new approach to studying flow and transfer of mobile DNA at an unprecedented resolution.

## Methods

### Strains

The recording strain (EcRec) was BL21 (NEB C2530H) with the pRec ΔlacI plasmid (Addgene #104575)^28^. Clinical *E. coli* isolates were a kind gift from Dr. Kristian Schønning, Hvidovre Hospital, Denmark. See Supplementary Data 6 for full overview of donor strains.

### Defined recordings

All strains were grown in LB medium with appropriate antibiotics and washed in PBS prior to recording. In all recordings an overnight culture of the recording strain was diluted 1:50 and grown for one hour, then anhydrotetracycline (ATc) was added to a final concentration of 100 ng/mL and the strain was incubated for another hour. Next, the recording strain and the donor strain were washed to remove antibiotics and resuspended in LB + aTc 100 ng/ml. The recording strain and donor strain were mixed 1:1 at OD600 = 0.5, except in the ratio experiment (Supplementary Methods) where strains were mixed in the ratios described in the figure. After mixing, the mixture was spotted on LB agar + 100 ng/mL aTc. Plates were incubated for 6 h at 37 °C. At the end of a recording, the cells were scraped off the plate and resuspended in 100 µl PBS and heat inactivated at 95 °C. for 3 min, subsequently they were stored at −20 °C until sequencing analysis.

### Recording of phage M13

Phage particles were generated by electroporating phage M13 DNA (NEB M13KE) into *E. coli* K603 and growing the strain to saturation in 10 ml LB followed by sterile filtration of the supernatant. The number of plaque forming units (pfu) was determined using the NEB protocol ‘M13 Titer Protocol’ (https://www.neb.com/protocols/2014/05/08/m13-titer-protocol) using EcRec/F’ as the indicator strain. Recording of M13 was done in LB with pre-induced EcRec/F’ at a concentration of 10^6^/ml and adding M13 at a multiplicity of infection (MOI) of 100. Recording was carried out for 24 h.

### Fecal recordings

The donor strain was prepared as described above. All fecal recordings were performed on fresh fecal samples (collected within 24 h of the recording). For each sample ~0.5 g were washed 2 times in 1 ml PBS and finally resuspended in 100 µl LB + 100 ng/ml ATc. The washed fecal sample was mixed with a 100 µl resuspension of 1 ml OD600 = 0.5 of the recording strain. From this mixture 50 µl was plated on LB agar + 100 ng/ml ATc and incubated for 24 h at 37 °C aerobically. Subsequently, the samples were processed as described above.

### Ethical review

The protocol for the collection of human samples and data was approved by the Columbia University Medical Center Institutional Review Board with a waiver of informed consent (IRB AAAR9489). Residual (waste) fecal specimens were used following standard clinical testing, and anonymized data was retrieved retrospectively.

### Array sequencing

CRISPR arrays were sequenced utilizing our established sequencing pipeline^28^ with minor modification. Briefly, DNA from cells was obtained by enzymatic and heat lysis, barcoded PCR amplification of CRISPR arrays was performed samples were pooled and sequencing was performed on the Illumina MiSeq platform (MiSeq v2 50 cycle, MiSeq v2 300 cycle or MiSeq v3 150 cycle kits) with additional spike-in of custom sequencing primers (for primer list see ref. ^28^). In addition, to enrich for expanded spacers, double gel extraction of expanded spacer bands on an E-gel EX Agarose Gel 2% was performed on pooled libraries. An overview of sequencing runs and sample statistics can be found in Supplementary Data 1, 2, 5

### Data processing

Spacers were extracted utilizing our established spacer extraction pipeline; code can be accessed at https://github.com/ravisheth/trace. Extracted spacers were filtered against the genome of the recording strain (quality filtered reads from sequencing of the same EcRec BL21/pRecΔlacI) using a two-step process using USEARCH v10.0.240 ref. ^45^. First spacers were filtered using a database of word size 8, then all non-hit spacers were collected and filtered against the same database using word size 5 (e.g., ‘usearch -usearch_global -id 0.8 -query_cov 0.8 -top_hit_only -maxrejects 0 -strand both -uc out.uc’). Subsequently the identified exogenous spacers were uniqued e.g.,(‘usearch -fastx_uniques -fastaout centroids.fa -sizeout’). The unique exogenous spacers was utilized in all subsequent spacer mapping performed with BLAST 2.7.1 + (‘blastn -db -query -perc_identity 90 -max_target_seqs 500000000 -task blastn -word_size 10 -outfmt “6 std sstrand qlen slen”). The output of BLAST was filtered to ensure 95% identity and 95% coverage of the query spacer. An example of the processing workflow can be seen in Supplementary Methods. Data analysis was performed in R^46^ using ggplot2 ref. ^47^ and CLC main workbench (www.qiagenbioinformatics.com).

### Reference databases

The following reference databases were used to identify the source of the acquired spacers: Prokaryotic RefSeq Genomes from January 2018; ftp://ftp.ncbi.nlm.nih.gov/genomes/refseq/bacteria/. Viral RefSeq Genomes from January 2018; ftp://ftp.ncbi.nlm.nih.gov/genomes/refseq/viral/. A custom plasmid database was created using the following search criteria in NCBI GenBank nucleotide database from January 2018; ‘plasmid[TI]’, then summary file was downloaded and parsed to get accession numbers of all circular elements:

‘grep -A1 ‘bp circular DNA’ summary.txt | grep -v ‘bp circular DNA’ | grep -v ‘\-\-‘ | cut -d’ ‘ -f1 > output.txt’ which were subsequently retrieved with NCBI batch (https://www.ncbi.nlm.nih.gov/sites/batchentrez).

### 16 S rRNA sequencing

16 S rRNA sequencing was performed utilizing our established sequencing pipeline; detailed methods can be found in our previous publication^48^. Briefly, genomic DNA (gDNA) was extracted with a protocol utilizing the Qiagen MagAttract PowerMicrobiome DNA/RNA kit (Qiagen 27500-4-EP). Samples were bead beat with 0.1 mm Zirconia Silica Beads (Biospec 11079101Z) for a total of ten minutes (Biospec 1001); the Qiagen kit protocol was followed but at reduced volumes on a Biomek 4000 liquid handling robot. The resulting gDNA was subjected to 16 S V4 amplicon sequencing utilizing custom barcoded primers^49^ and NEBNext Q5 Hot Start HiFi Master Mix (NEB M0543L). Resulting PCR products were quantified and pooled on a Biomek 4000 robot and sequenced utilizing an Illumina MiSeq V2 300 cycle kit. The sequencing data was analyzed using USEARCH 10.0.240 ref. ^45^; reads were merged (-fastq_mergepairs), filtered (-fastq_filter -fastq_maxee 1.0 -fastq_minlen 240), and 100% ZOTUs were generated (-unoise3) and OTU table created (-otutab). Taxonomy was assigned to ZOTUs using the RDP classifier^50^. The OTU table was rarefied to 1000 reads per sample before analysis.

### Whole genome and shotgun metagenomic sequencing

The recording strain BL21/pRec along with all donor strains were subjected to whole genome sequencing (Supplementary Data 6) and clinical samples were subjected to shotgun metagenomic sequencing (Supplementary Data 5). gDNA was extracted from individual isolates or fecal samples utilizing the gDNA extraction pipeline detailed above. Sequencing preparation followed a published protocol for low-volume Nextera library preparation^51^. Barcoded samples were pooled and sequencing was performed on the Illumina MiSeq (2 × 150 reads), Illumina NextSeq (2 × 75 reads) or Illumina HiSeq X platform (2 × 150 reads). Adapters were trimmed utilizing Trimmomatic^52^. Draft assemblies for the donor strains were conducted using SPAdes utilizing the --careful flag^53^. Metagenomes were assembled with SPAdes utilizing the --meta flag. Raw metagenomic reads were mapped to the refseq viral database as well as the plasmid database using bwa mem^54^.

The donor strain Ec70 was further sequenced utilizing the Oxford MinION platform; genomic DNA was extracted with a Gentra Puregene kit (Qiagen), prepared for sequencing utilizing the RAD004 kit and sequenced on a single R9.4.1 flow cell. For this strain, hybrid assembly of the genome and individual plasmids was conducted utilizing UniCycler^55^. See Supplementary Data 6 for genome sequencing information and Supplementary Data 5 for metagenome sequencing information.

### Reporting summary

Further information on research design is available in the Nature Research Reporting Summary linked to this article.

## Supplementary information

Supplementary Information

Description of Additional Supplementary Files

Supplementary Data 1

Supplementary Data 2

Supplementary Data 3

Supplementary Data 4

Supplementary Data 5

Supplementary Data 6

Reporting Summary

## Data Availability

Assembled genomes, metagenomic reads, and CRISPR array sequencing data is deposited under bioproject number PRJNA594543. All other relevant data are available from the corresponding author upon request.

## Code availability

CRISPR array spacer extraction software can be accessed at https://github.com/ravisheth/trace. Code for the subsequent filtering and spacer identification pipeline is described in the Supplementary Methods.
