# Supplementary material for: Recording mobile DNA in the gut microbiota using an Escherichia coli CRISPR-Cas spacer acquisition platform
Source: Nat Commun. 2020 Jan 7;11:95. doi: 10.1038/s41467-019-14012-5 (PMC6946703; doi:10.1038/s41467-019-14012-5)
Supplement: Supplementary file 1 — Supplementary Information [file 41467_2019_14012_MOESM1_ESM.pdf]

Supplementary Figures

Recording mobile DNA in the gut microbiota using an *Escherichia coli* CRISPR-Cas spacer acquisition platform

Munck and Sheth *et al.*

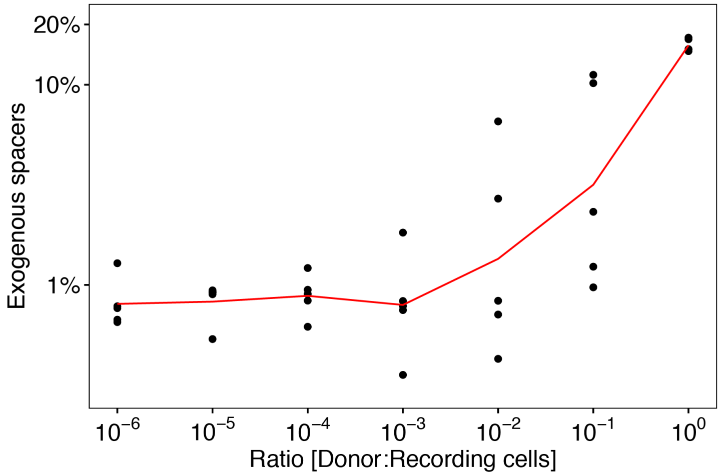

Supplementary Figure 1: Effect of donor ration of spacer acquisition.

Donor and EcRec was mixed in ratios from 10^{-6} – 10^{0} and spotted on LB agar. Recording was carried out for 6 hours.

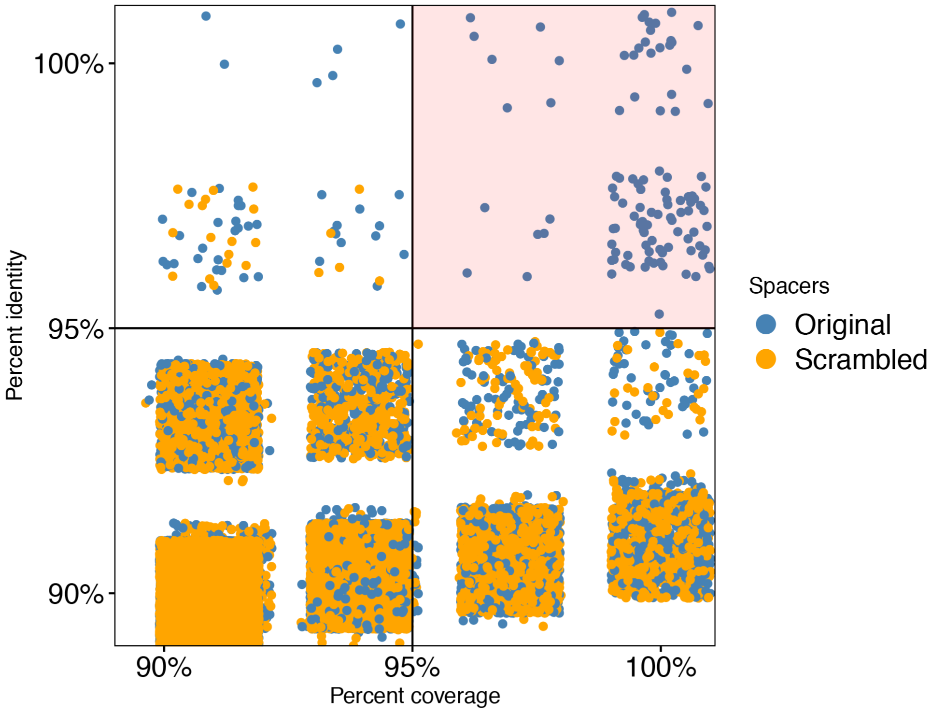

Supplementary Figure 2. Identifying mapping cutoff.
To identify cutoff for spacer mapping to databases of potential donors (e.g. Genbank nt) the recorded spacers from the E. coli FS1290/RP4 recording were scrambled by random reordering the sequence. Both the original and the scrambled spacers were mapped to the Genbank nt database using BLAST. We identified cutoffs of >=95% identity and coverage as resulting in reliable assignment of spacers (pink space). Each data point represent a unique spacer sequence.

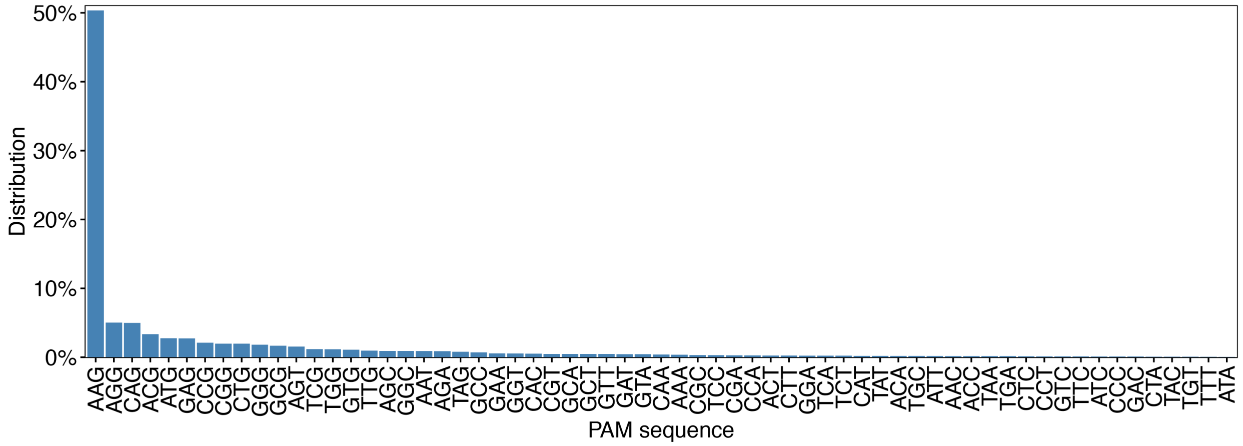

Supplementary Figure 3. Distribution of protospacer adjacent motifs (PAM).
PAM sequences were extracted for all spacers from the *E. coli* FS1290/RP4 mapping. The distribution shows a clear preference for spacers with the canonical AAG sequence.

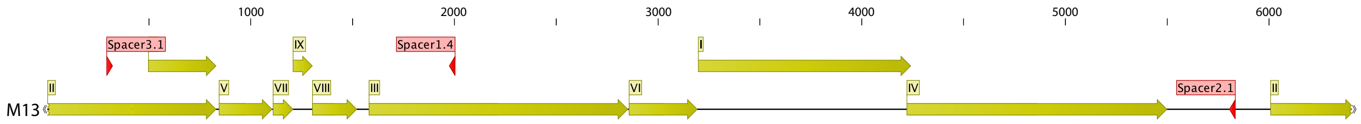

Supplementary Figure 4. Spacers matches to phage M13.
Mapping of spacers to the genome of phage M13 (NC_003281). Each unique spacer is marked with red and labelled with a unique number followed by the number of spacers representing the unique match.

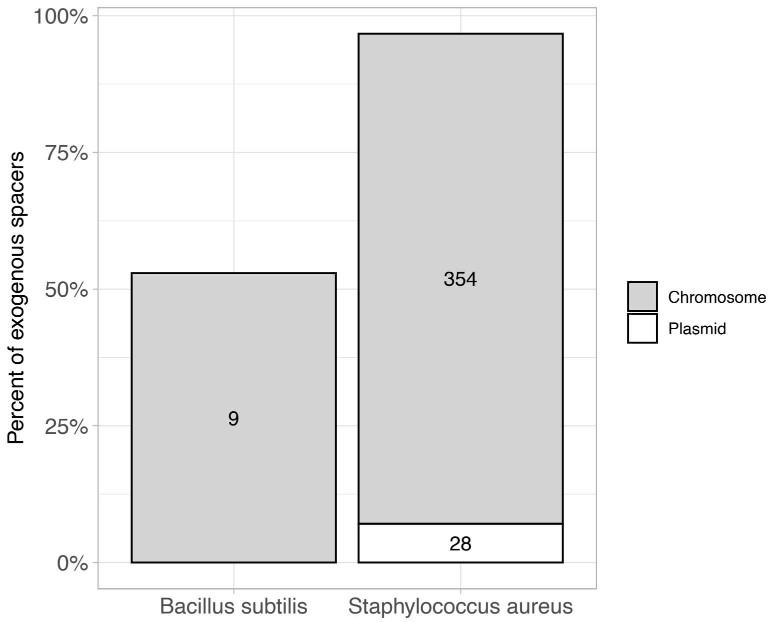

Supplementary Figure 5. Detecting spacers from electroporated Gram positive DNA. Purified plasmids pGO400 and pSL20 from *S. aureus* and *B. subtilis*, respectively, were electroporated into induced EcRec. EcRec was recovered for 24 hours with induction of cas1/2. All exogenous spacers were mapped to the refseq database. For each sample the percent of exogenous spacers mapping to the relevant host is shown with the actual spacer count noted inside each category. Despite using purified plasmid, the majority of spacers mapped to the genomes of *B. subtilis* and *S. aureus* respectively, suggesting that the plasmid prep contained substantial chromosomal contamination.

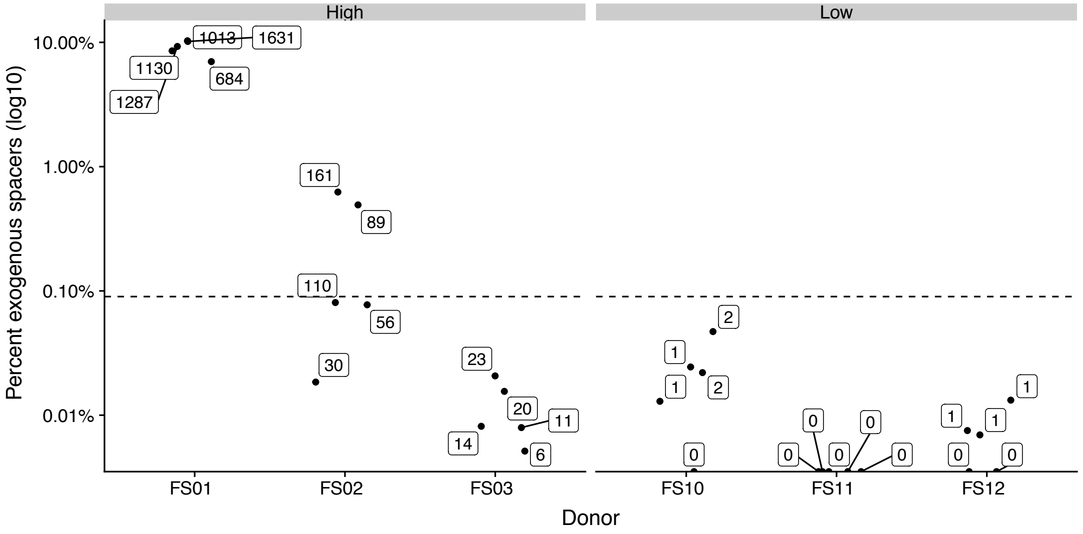

Supplementary Figure 6. Reproducibility of fecal recordings.
Repeat recordings were performed in five replicates in three samples with initial high number of exogenous spacers (FS01-03) and three samples with a low number of exogenous spacers (FS10-12). The overall degree of reproducibility is high, with a similar number of relative spacer adaptations within each sample. For FS02 and FS03 the percent exogenous spacers are lower than in the initial recording, with most replicated falling below the inclusion threshold (dashed line). We speculate that this might be caused by the freeze/thawing of the fecal samples between the two recordings. We also note, that array amplification from the low exogenous samples is less efficient, suggesting that the recording strain might be inhibited or killed in these samples.

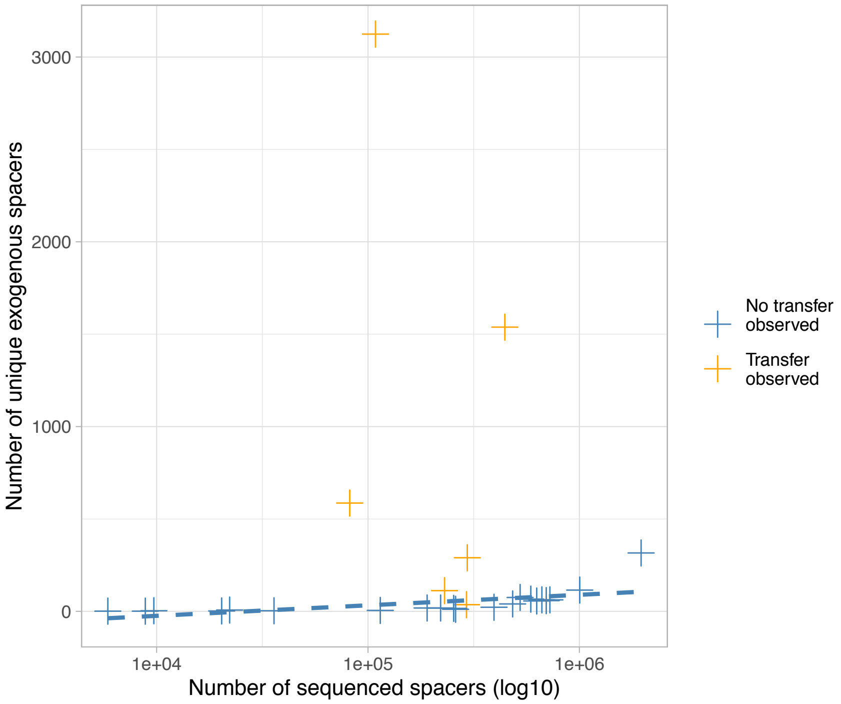

Supplementary Figure 7. Correlation between sequencing depth and exogenous spacers. For the six samples with observed transfer (Orange) there is no correlation between sequencing depth and number of exogenous spacers. In contrast, in the samples with no observed transfer (blue), there is a correlation between sequencing depth and number of exogenous spacers, probably driven by sequencing noise.

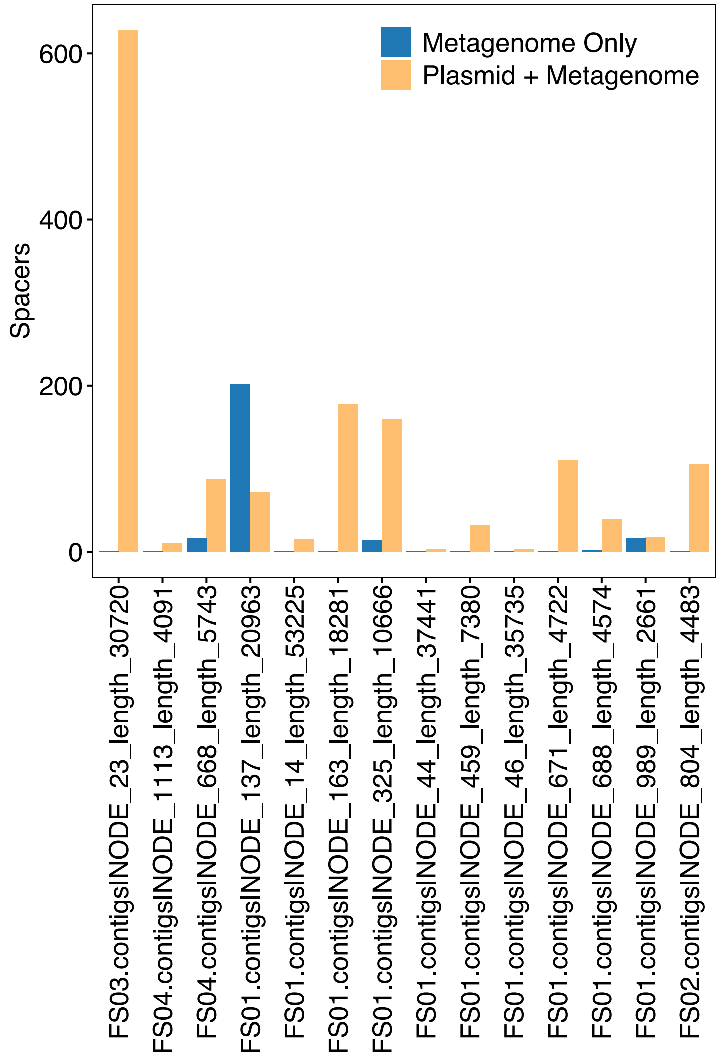

Supplementary Figure 8. Contigs with metagenome-only spacers.
Metagenomic contigs >500 bp that have at least two spacers mapping that do not map to the plasmid database. Shown is the number of spacers that only map to a metagenomic contig (blue bars) as well as spacers that map to both a metagenomic contig and a plasmid in the custom plasmid database. In all cases but one, most spacers mapping to a metagenomic contig also map to a plasmid contig indicating that the transferred element is known. However, in FS01 Node_137 the majority of the spacers only match to the metagenomic contig suggesting that most of this transferred element is not commonly found in plasmids.

Supplementary Methods

Recording mobile DNA in the gut microbiota using an *Escherichia coli* CRISPR-Cas spacer acquisition platform

Munck and Sheth *et al.*

Spacer analysis workflow.
Spacers are first extracted and processed with the workflow below to remove endogenous spacers and then match identified exogenous spacers to relevant databases.

1. **Extract spacers from raw sequencing files from Illumina instrument**

   Our previously published spacer extraction pipeline was utilized; code is available at https://github.com/ravisheth/trace

2. **Search spacers against the EcRec/pRec reference genome, using database with word size 8**

   ```
   usearch -usearch_global input.fa -db ref.reads.udb.fasta.8.udb -id 0.8 -query_cov 0.8 -
   top_hit_only -maxrejects 0 -strand both -uc out.uc
   ```

3. **Compile a fasta file with sequences not mapping to the word size 8 database**

   ```
   #Get the ids of the non hits
   find ./ -type f -name 'out.uc' | while read F
   do
     awk -F$'\t' '$1=="N" { print $9 }' ${F} > ${F}.exogenous.id.txt
   done
   #Compile a fasta file with the non hit sequences
   find ./ -type f -name 'input.fa' | while read F
   do
     grep -F -A1 -f ${F}.uc.exogenous.id.txt ${F} | sed '/^--/d' > ${F}.exogenous.ws8.fa
   done
   ```

4. **Search remaining spacers against the EcRec/pRec reference genome, using database with word size 5**

   ```
   usearch -usearch_global exogenous.ws8.fa -db ref.reads.udb.fasta.5.udb -id 0.8 -query_cov 0.8 -
   top_hit_only -maxrejects 0 -strand both -uc out.uc
   ```

5. **Compile a fasta file with sequences not mapping to word size 8 or word size 5 databases (i.e. exogenous spacers)**

   ```
   #Finally get all the exogenous spacers
   find ./ -type f -name 'out.uc' | while read F
   do
     awk -F$'\t' '$1=="N" { print $9 }' ${F} > ${F}.exogenous.id.txt
   done
   #Compile a fasta file with the non hit sequences
   find ./ -type f -name 'input.fa ' | while read F
   do
     grep -F -A1 -f ${F}.exogenous.ws8.fa.uc.exogenous.id.txt ${F} | sed '/^--/d' > ${F}.exogenous.fa
   done
   ```

6. **Cluster exogenous spacers**

   ```
   for file in *.exogenous.fa
   do
     usearch -fastx_uniques $file -fastaout $file.centroids.fa -sizeout
   ```

```
done
```

7. BLAST unique exogenous spacers against desired database

```
blastn -db RefSeqJan2018 -query centroids.fa -perc_identity 90 -max_target_seqs 500000000 -task blastn -word_size 10 -num_threads 5 -outfmt "6 std sstrand qlen slen" -out centroids.refseq.hits.txt
```
