# Supplementary material for: Recording mobile DNA in the gut microbiota using an Escherichia coli CRISPR-Cas spacer acquisition platform
Source: Nat Commun. 2020 Jan 7;11:95. doi: 10.1038/s41467-019-14012-5 (PMC6946703; doi:10.1038/s41467-019-14012-5)
Supplement: Supplementary file 2 — Description of Additional Supplementary Files [file 41467_2019_14012_MOESM2_ESM.docx]

File Name: **Supplementary Data 1**
Description: Overview of sequenced spacers, time resolved, no gel-extraction.

File Name: **Supplementary Data 2**

Description: Overview of sequenced spacers, with gel extraction.

File Name: **Supplementary Data 3**

Description: Identification of plasmids in the eight clinical E. coli. Plasmids are identified via blast to the Plasmid Finder database using thresholds >=90% identity and coverage.

File Name: **Supplementary Data 4**

Description: Overlapping annotations for the 5357 unique spacers in the recording rom the human fecal samples. Annotations are extracted from spacer matched to the NCBI Refseq genomes database.

File Name: **Supplementary Data 5**

Description: Shotgun metagenome sequencing and assembly statistics (peformed with SPAdes and statistics calculated with quast).

File Name: **Supplementary Data 6**

Description: Sequenced strains.
